# Drug-resistant HIV-1 protease regains functional dynamics through cleavage site coevolution

**DOI:** 10.1111/eva.12241

**Published:** 2015-01-11

**Authors:** Nevra Özer, Ayşegül Özen, Celia A Schiffer, Türkan Haliloğlu

**Affiliations:** 1 Polymer Research Center and Chemical Engineering Department, Bogazici UniversityBebek, Istanbul, Turkey; 2 Department of Biochemistry and Molecular Pharmacology, University of Massachusetts Medical SchoolWorcester, MA, USA

**Keywords:** coevolution, elastic network model, fluctuations, HIV-1 protease

## Abstract

Drug resistance is caused by mutations that change the balance of recognition favoring substrate cleavage over inhibitor binding. Here, a structural dynamics perspective of the regained wild-type functioning in mutant HIV-1 proteases with coevolution of the natural substrates is provided. The collective dynamics of mutant structures of the protease bound to p1-p6 and NC-p1 substrates are assessed using the Anisotropic Network Model (ANM). The drug-induced protease mutations perturb the mechanistically crucial hinge axes that involve key sites for substrate binding and dimerization and mainly coordinate the intrinsic dynamics. Yet with substrate coevolution, while the wild-type dynamic behavior is restored in both p1-p6 (^LP^^1′F^p1-p6_D30N/N88D_) and NC-p1 (^AP^^2^^V^NC-p1_V82A_) bound proteases, the dynamic behavior of the NC-p1 bound protease variants (NC-p1_V82A_ and ^AP^^2^^V^NC-p1_V82A_) rather resemble those of the proteases bound to the other substrates, which is consistent with experimental studies. The orientational variations of residue fluctuations along the hinge axes in mutant structures justify the existence of coevolution in p1-p6 and NC-p1 substrates, that is, the dynamic behavior of hinge residues should contribute to the interdependent nature of substrate recognition. Overall, this study aids in the understanding of the structural dynamics basis of drug resistance and evolutionary optimization in the HIV-1 protease system.

## Introduction

Protein interactions mediate the function of biological systems, where the evolution of interactions is important to understand the functional mechanism in act (Juan et al. 2008; Lovell and Robertson 2010). Evolutionary signals are generated either by whole-sequence evolution or by site-specific coevolution (Lovell and Robertson 2010). Coevolution can be defined as a reciprocal change in one site affecting the selection pressure at another site allowing for adaptation (Thompson 1994). This can occur as either an intramolecular or an intermolecular process, where coevolution arises from the evolutionary interaction between sites within a single molecule in the former, and the latter is due to co-adaptation as a result of the evolutionary interaction between different molecules (Juan et al. 2008; Lovell and Robertson 2010).

Understanding evolution within the complex relationship between sequence, structure and function for a particular phenotype is quite limited (Xia and Levitt 2004; Tomatis et al. 2008). Selective pressures for evolvability should act at both structural and dynamics levels, where the sequence divergence is constrained by the conservation of structural features and further by the conservation of functional motion. Thus, the functional importance of protein dynamics should be credited for the sequence evolution (Maguid et al. 2008; Juan et al. 2008). The structural flexibility and plasticity of proteins are imperative in performing their biological functions, especially in molecular recognition (Teague 2003; Gerstein and Echols 2004; Marianayagam and Jackson 2005; Friedland et al. 2009; Ramanathan and Agarwal 2011; Mittal et al. 2012). The mechanism of this recognition between proteins and ligands is probably predefined by the rules encoded in the protein structure and dynamics, the detailed knowledge of which would be useful in design and engineering of drugs. Evolvability and natural variation are also correlated with drug resistance, which is a central problem in drug design for many diseases (Earl and Deem 2004; Creavin 2004; Berkhout and Sanders 2005; Nalam and Schiffer 2008). Influenza, tuberculosis, malaria, cancer, and HIV/AIDS are some of the important examples of diseases that confront drug resistance.

HIV-1 protease is an effective therapeutic target of the most effective antiviral drugs for the treatment of HIV-1 infection (Prabu-Jeyabalan et al. 2002). The protease is a symmetric homodimer containing a single active site formed at the dimer interface by two conserved catalytic aspartic acid residues, one from each monomer, and covered by two flexible flaps (Wlodawer and Erickson 1993). The enzyme recognizes substrate sites on the Gag and Gag-Pro-Pol polyproteins which are asymmetric in both size and charge around the cleavage site, while the currently prescribed inhibitors are relatively symmetric. Yet with the drug-resistant mutations on the protease, the affinity for inhibitors is lowered while efficient processing is still maintained and the structure reassumes a drastic asymmetry (Prabu-Jeyabalan et al. 2004). Thus, substrate specificity should be based on a conserved shape rather than a particular amino acid sequence. This is explained by a consensus volume of substrates recognized by the protease, defined as the ‘substrate envelope’, which not only explains specificity but also has significant implications for drug resistance and substrate coevolution (Kolli et al. 2009). The envelope is achieved by packing of the substrate residues which makes the substrate recognition interdependent (Prabu-Jeyabalan et al. 2002; King et al. 2004). This interdependency implies that a drug-resistant protease mutation that causes unfavorable interactions with one substrate position is often compensated by a mutation at another position within the substrate sequence. Two examples of this evolutionary interplay between substrate and enzyme are the coevolution of p1-p6 substrate cleavage site with D30N/N88D protease mutations (Bally et al. 2000) and the coevolution of NC-p1 substrate cleavage site with V82A protease mutation (Doyon et al. 1996). Furthermore, cleavages at these sites of Gag, which have the most significant polymorphism among all HIV-1 substrate sites, are known to be rate limiting steps in polyprotein processing (Tözser et al. 1991; Feher et al. 2002).

Most drug-resistant mutations within the protease active site occur where the inhibitors protrude beyond the substrate envelope and contact the protease (King et al. 2004). Those protease mutations may be associated with other mutations in the substrate sites that extend beyond the substrate envelope and/or with other mutations in the viable protease (Kolli et al. 2006). Substrate dynamics was later incorporated into the substrate envelope, where p1-p6 and NC-p1 are shown to be two of the three most dynamic substrates (Ozen et al. 2011). Dynamic substrates exhibit a worse fit within this dynamic substrate envelope as they sample a wider conformational space resulting in a greater deviation from the substrate envelope. Accordingly, the dynamic p1-p6 and NC-p1 substrates protrude beyond the dynamic envelope more than expected based on their molecular volume compared to the other substrates (Ozen et al. 2011). Thus, the compensatory mutations in these cleavage sites optimize the portion of the substrate volume that stays within the dynamic substrate envelope. In the presence of D30N/N88D mutations in the protease, the coevolutionary mutation LP1′F at the p1-p6 cleavage site provides a better fit within the dynamic substrate envelope. Similarly, in NC-p1, the compensatory AP2V substitution occurs in the presence of V82A mutation in the protease. The interdependency between the changes in the substrate sequence in response to the drug-induced protease mutations can be explained by the energetic fitness of the sequences to the structural space of HIV-1 protease. This fitness possibly implies a conservation for the dynamic behavior of the residues in the HIV-1 protease-substrate/inhibitor complex system. With this approach, the structural basis of drug resistance, that is, the effect of structural and dynamic constraints on the sequence evolution (Liu and Bahar 2012; Gerek et al. 2013), could further be clarified. In this study, we investigate the dynamics to understand the mechanism that results in the dynamic substrate envelope which was validated as the substrate recognition motif for HIV-1 protease.

Significant structural and functional features of biomolecular complexes can be elucidated by the detailed analysis of fluctuations around their native states (Bahar et al. 2010). When dynamics is decomposed into a collection of modes of motion, the cooperative low frequency/large amplitude modes have been shown to be significantly correlated with the biological function (Nicolay and Sanejouand 2006). The principal component analysis (PCA) is a computational approach to extract the collective behavior from the fluctuations observed in molecular dynamics (MD) trajectories (Tournier and Smith 2003). The cooperative motions can alternatively be studied by normal mode analysis (Ma 2005; Cui and Bahar 2005). Elastic network models have been well-accepted for studying the large-scale motion of protein structures in recent years (Chennubhotla et al. 2005; Nicolay and Sanejouand 2006; Bahar et al. 2010; Gniewek et al. 2012). Despite the simplicity of this approach, the application of the elastic network models such as the Gaussian Network Model (GNM) (Bahar et al. 1997; Haliloglu et al. 1997) and the Anisotropic Network Model (ANM) (Atilgan et al. 2001) to the HIV-1 protease system have also produced results that are highly in accord with those of both experimental studies and MD simulations (Bahar et al. 1998; Zoete et al. 2002; Kurt et al. 2003; Micheletti et al. 2004; Hamacher and McCammon 2006; Yang et al. 2008; Hamacher 2010). The computational studies on the structural dynamics of HIV-1 protease suggest that understanding the dynamic behavior of the enzyme is crucial for its intrinsic flexibility and function (Perryman et al. 2004; Hornak and Simmerling 2007; Ozer et al. 2010).

In protein-ligand interactions, the ligand prefers the conformations that best match its structural and dynamic behavior among those intrinsically accessible to the unbound protein (Bakan and Bahar 2009). The conformational changes experimentally observed in the enzymes by binding a broad range of ligands can be predicted by the most cooperative lowest frequency modes of motion by ANM, where the hinges are the key mechanistic regions of the structure that control the conformational ensemble. Hinge motion has been shown to be an important mechanism that underlies the functional conformational changes, and catalytic residues tend to be positioned near the hinge regions that are unique for particular architectures (Yang and Bahar 2005). The elastic distortions of these dynamically important hinge residues, which serve as central hubs, effectively trigger the correlated fluctuations of a large number of residues (Zheng and Brooks 2005). Thus, the dynamic behavior of the hinge axes should mainly determine the flexibility and intrinsic dynamics of the structure. In our recent work, the ANM analysis of the fluctuations of the bound HIV-1 protease structures demonstrated that the hinge residues of the most cooperative modes display variation in their fluctuations depending on the bound substrate (Ozer et al. 2010). Further, flexible substrates adapt to the conformational changes of the protease better than the conformationally and dynamically restricted inhibitors, implying the rationale for more diverse inhibitors. Here, to study the dynamic behavior that accompany coevolution in the HIV-1 protease system, fluctuations of the wild-type, mutant, and coevolved structures of two protease-substrate complexes, namely the p1-p6 and NC-p1 complexes, are analyzed by ANM. The results put forward the motive for the evolutionary optimization of the sequences of HIV-1 protease-substrate complex from a mechanistic functional dynamics perspective.

## Materials and methods

### Nomenclature

Throughout this article, the wild-type HIV-1 protease (WT), the HIV-1 protease mutants (D30N, D30N/N88D, or V82A), and the cleavage site (LP1′F or AP2V) variants in a protease-substrate complex are designated by a subscript and a superscript to the name of the cleavage site. For example, ^LP1′F^p1-p6_D30N_ denotes a complex of D30N protease variant with the LP1′F mutant of the p1-p6 cleavage site, where LP1′F refers to a Leu-to-Phe mutation at P1′ position of the cleavage site. The substrate residues on the amino-terminal side of the scissile bond and the protease monomer on that side are termed as unprimed; whereas, those on the carboxy-terminal side of the scissile bond are termed as primed.

### Structures

The wild-type structures are the crystal structures of HIV-1 protease in complex with its natural substrates downloaded from the Protein Data Bank (PDB) (Bernstein et al. 1977; Prabu-Jeyabalan et al. 2002, 2004). The structures of p1-p6_D30N_, p1-p6_D30N/N88D_, ^LP1′F^p1-p6_D30N/N88D_, NC-p1_V82A_, and ^AP2V^NC-p1_V82A_ variants are modeled *in silico* based on their wild-type structures and simulated by MD simulations. The details of the modeling and MD simulation protocols were described elsewhere (Ozen et al. 2012). The representative conformations are selected by clustering the MD sampled conformations.

### Cluster analysis on MD simulation trajectories

A representative set of conformations of all wild-type protease-substrate complexes, p1-p6_D30N_, p1-p6_D30N/N88D_, ^LP1′F^p1-p6_D30N/N88D_, NC-p1_V82A_, and ^AP2V^NC-p1_V82A_, generated from MD simulation trajectories of eleven ns production phase are clustered separately to group the ‘redundant’ conformations and examine the unique conformers. For the modeled mutant structures that were run for fifteen *n*s, the clusters of the endmost snapshots of eleven ns are used to allow selection of MD-relaxed structures. The similarity measure to group the MD sampled conformations is root-mean-square deviation (RMSD) in this study. MMTSB Toolset's (Feig et al. 2004) *kclust* utility that uses the k-means clustering is used to perform conformational clustering. The convergence of the simulations is judged by the relative populations of clusters as even fairly long MD trajectories may not be converged for flexible systems (Lyman and Zuckerman 2006). The number of clusters depends on the cutoff value of RMSD (cluster radius); as RMSD cutoff increases, less number of clusters are found by the algorithm. With a total of 550 structures for each HIV-1 protease-substrate complex within eleven ns, the cluster radius is set as 1.3 Å after various trials. The number of clusters obtained hereby and the percentage of all the clusters can be seen on Table1. The percentage of largest clusters varies between 35% and 86% while that of the largest two clusters in total varies between 67% and 100%. Then, the representative structures of the largest clusters are further analyzed by ANM. To test the convergence in the dynamic behavior observed, the representative members of the second largest clusters are also analyzed by ANM and the results are compared with those of the largest clusters.

**Table 1:** Percentage of the clusters of molecular dynamics (MD) sampled structures

	p1-p6_WT_	p1-p6_D30N/N88D_	^LP1′F^p1-p6_D30N/N88D_	NC-p1_WT_	NC-p1_V82A_	^AP2V^NC-p1_V82A_	MA-CA_WT_	CA-p2_WT_	p2-NC_WT_	RT-RH_WT_	RH-IN_WT_
Cluster 1	50	57	41	37	51	35	40	68	65	86	49
Cluster 2	25	36	32	34	39	32	29	23	23	14	42
Cluster 3	16	7	17	17	10	15	22	9	12		9
Cluster 4	5		10	12		12	9				
Cluster 5	4					6					

### Anisotropic Network Model

The simple elastic network models are originally introduced by Tirion (Tirion 1996), where the complex vibrational properties of macromolecular systems are reproduced by a model using a single uniform harmonic potential. GNM (Bahar et al. 1997; Haliloglu et al. 1997) and ANM (Atilgan et al. 2001) are the two models that have widely been used in recent years.

Anisotropic Network Model (Atilgan et al. 2001) predicts the directionalities and magnitudes of the motions of protein structures around their equilibrium states by a harmonic vibrational analysis. The conformations that describe the fluctuations of residues from the average in the principal directions of motion are generated using the elastic network formed by connecting all neighboring heavy atoms. The total potential energy for a system of *N* nodes is the summation over all harmonic interactions of close-neighboring (*i*,*j*) pairs calculated as:

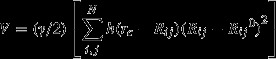

*γ* is the harmonic force constant, and *R*_*ij*_ is the instantaneous distance, and 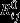 is the equilibrium distance between sites *i* and *j* in the native structure. *h*(*r*_*c*_*−R*_*ij*_) is the Heaviside step function which is 1 if (*r*_*c*_*−R*_*ij*_) ≥0 and zero otherwise. *r*_*c*_, the cutoff distance, is taken as 9 Å, which has successfully been used to account for inter-residue interactions in the all-atom structure model of the HIV-1 protease system (Ozer et al. 2010).

The Hessian matrix ***H*** is a 3*N* × 3*N* symmetric matrix, which holds the anisotropic information regarding the orientation of nodes *i, j*. ***H*** is composed of *N* × *N* super elements ***H***_*ij*_ each of size 3 × 3 given by the second derivatives of the potential *V*. An orthogonal transformation of the real symmetric Hessian matrix gives the normal modes of the elastic network with 3*N *− 6 nonzero eigenvalues *λ*_*i*_ and corresponding eigenvectors **u**_*i*_.

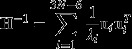


The fluctuations of nodes are used to construct and explicitly view pairs of alternative conformations sampled in the individual modes, simply by adding the fluctuation vectors to the equilibrium position vectors in the respective modes. The mode shapes exhibit the distribution of mobility among residues driven by different frequency modes, where the minima correspond to hinge regions.

### Orientational correlation analysis

The orientational correlation between the fluctuations in different structures is assessed by the calculation of the inner product of the fluctuation vectors. That is, the structures are superimposed, and cosine of the angle between their fluctuation vectors is evaluated by a dot product calculation. The normalized correlation values range between 1 (perfect correlation, where the angle between the fluctuation vectors of the superimposed structures are 0°) and −1 (maximum variation in the fluctuation direction, where the angle between the fluctuation vectors of the superimposed structures are 180°). The orientational correlations are assessed on a residue basis in the two most cooperative modes of motion, to observe which residues' fluctuations differ between various protease-substrate complex structures. Absolute lower orientational correlation values indicate the residues that display larger variations in their fluctuations' directions.

## Results and discussion

The HIV-1 protease structures investigated in this study are listed on Table2. Details of the nomenclature used throughout the article are described in the Materials and methods section.

**Table 2:** HIV-1 protease-substrate complex structures used in the analyses

Wild-type structures (PDB code)	p1-p6_WT_ (1kjf)
	NC-p1_WT_ (1tsu)
	MA-CA_WT_ (1kj4)
	CA-p2_WT_ (1f7a)
	p2-NC_WT_ (1kj7)
	RT-RH_WT_ (1kjg)
	RH-IN_WT_ (1kjh)
Mutant structures	p1-p6_D30N_
	p1-p6_D30N/N88D_
	NC-p1_V82A_
Coevolved mutant structures	^LP1′F^p1-p6_D30N/N88D_
	^AP2V^NC-p1_V82A_

### Cooperative motion of protease and substrate

**Figure 1:**
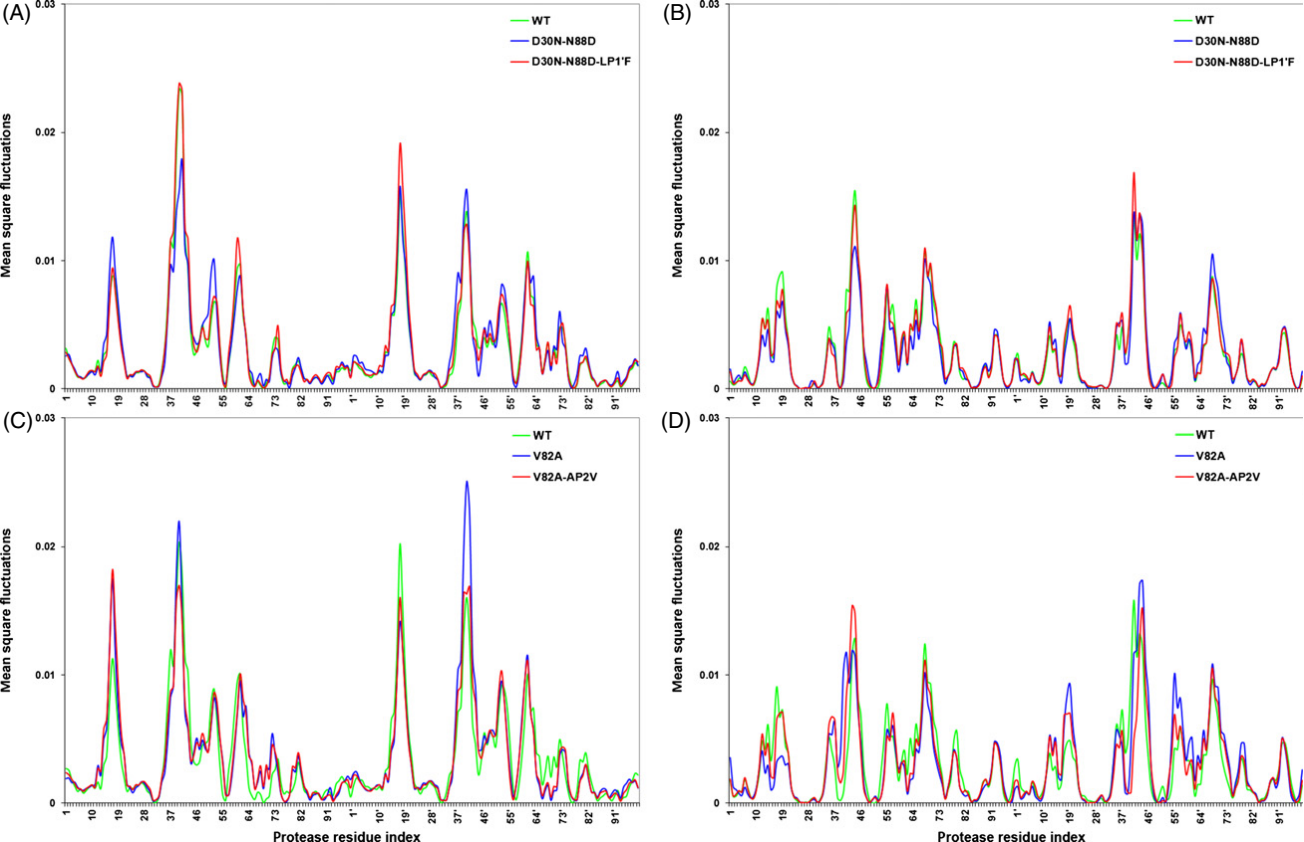
Mean-square fluctuations of the p1-p6 and NC-p1 bound protease complex structures in the first (A, C) and in the second (B, D) slowest Anisotropic Network Model (ANM) modes. The fluctuations are calculated for all the atoms in the structures, but for clearer representation, the fluctuations of C_*α*_ atoms are plotted, and the corresponding residue numbers are indicated on the *x*-axis.

The slow modes describe the most cooperative global motion of the HIV-1 protease-peptide complex structures and are likely associated with the enzymatic function of the protease (Kurt et al. 2003; Yang and Bahar 2005; Yang et al. 2008). The residue fluctuation profiles in the slowest two modes share a similar trend in the wild-type and mutant complexes investigated here (Fig.1), which is consistent with the earlier results on the wild-type, substrate- and inhibitor-bound protease structures (Ozer et al. 2010). The generality of this observation across substrate and inhibitor complexes of protease variants suggest that the hinge regions such as the dimerization region (res. 5–10), the active site (res. 25–27), the flap (res. 45–55), and the substrate cleft (res. 80–90) display the least fluctuations in their mean positions and coordinate the motion. The distribution of the mean-square fluctuations in p1-p6 and NC-p1 bound protease structures analyzed here displays that residues 56, 69, 78, 93 and residues 25–27, 49–51, 84, 97 are observed to be at the minima of the corresponding mode shapes, in the first (Fig.1A,C) and second (Fig.1B,D) slowest modes, respectively.

**Figure 2:**
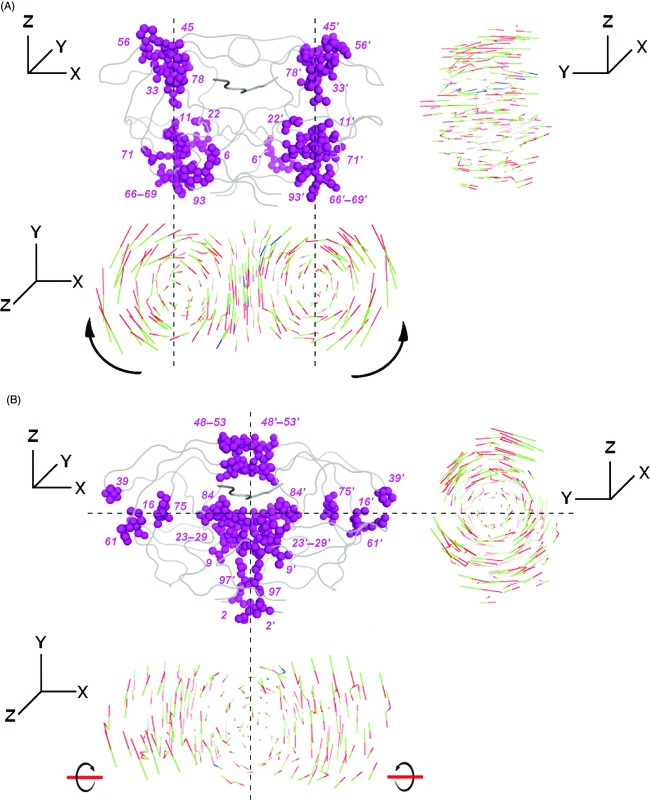
The regions of the orientational difference in the direction of fluctuations in the first (A) and second (B) slowest Anisotropic Network Model (ANM) modes. The least correlating residues in their fluctuations' directions between different complex structures of HIV-1 protease are displayed in magenta in the front view. Top and side views of the structure are shown, where the residue fluctuations in each mode are represented as moving between the conformations shown in green and red for the protease, and in green and blue for the peptide. The dashed lines indicate the hinge axes around which the monomers rotate. The coordinate system for the front, top, and side views is indicated next to the structures.

The motion of a ligand-bound HIV-1 protease is demonstrated for one representative structure in Fig.2. In the slowest mode (Fig.2A), the protease monomers rotate around two axes parallel to the *Z* direction, coupled with the peptide fluctuation along the *Y* direction. In second slowest mode (Fig.2B), the monomers rotate around two different axes parallel to *X* and *Z* directions and the peptide motion is significant in the terminal residues. The hinge axes in the corresponding modes are shown as dashed lines. As the functional conformational changes are mainly due to elastic distortions of the hinge residues that trigger the correlated fluctuations (Zheng and Brooks 2005), the dynamic behavior of the hinge axes, that is, the fluctuation of residues located at the hinge sites, should determine the flexibility and intrinsic dynamics of the bound protease. Here, the hinge regions suggested by the slowest mode mainly coordinate the intrachain cooperative motions along with the motion of the peptide, whereas those suggested by the second slowest mode are mostly responsible for the correlations across the dimerization interface (Fig.2).

### Deformation of hinge axes by protease mutations and coevolution: the orientational variations in the fluctuations of hinge residues

Among different complex structures of the wild-type protease, although the overall residue mobility profiles are similar (Fig.1), some variations in the amplitude and the orientation of the residue fluctuation vectors might be possible. The substrate stabilizes one of the several conformations accessible to the protease functionally and energetically favorable (from both thermodynamic and kinetic aspects). For example, the protease's interaction with a specific substrate should corroborate the substrate kinetics, that is, the rate of cleavage. Structural variations across substrate complexes should be reflected in specific recognition of substrates, so should be the difference in their dynamic behavior. This presumable difference is therefore investigated further by the orientational correlations between the residue fluctuations in mutant structures.

In the ANM analysis of the dynamics of the wild-type HIV-1 protease structures bound to its natural substrates, the highest orientational differences in the residue fluctuations and the largest extent of the asymmetry of the residue fluctuations in the two protease monomers were observed primarily along the hinge axes (Ozer et al. 2010). This suggests that there is a substrate-specific behavior implicated therein in relevant modes (Fig.2) by the fluctuations of hinge residues. Here, the premise is whether the specific dynamic interaction of each substrate with the protease should be conserved or not. To this end, the inquiry is whether or not the drug-induced protease mutations associated with the coevolution of the substrate would corroborate this specific interaction by the re-orientation of fluctuations of hinge residues back to those of the wild type. The analysis of the orientational correlations (see Materials and methods) of the residue fluctuations among the mutant and the coevolved mutant structures will provide a dynamic mechanistic perspective for the role of mutations.

#### Coevolution in p1-p6

**Figure 3:**
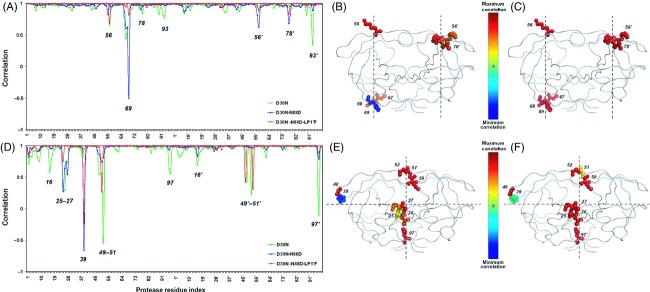
Orientational correlation of the protease residues' fluctuations of p1-p6_D30N_, p1-p6_D30N/N88D_, and ^LP^^1′F^p1-p6_D30N/N88D_ to those of p1-p6_WT_ in the first (A) and second (D) slowest modes. The residues that display the maximum variations between the directions of fluctuations are color coded according to the change in the correlations of p1-p6_D30N/N88D_ and ^LP^^1′F^p1-p6_D30N/N88D_ compared to p1-p6_WT_; (B) and (C) in the first slowest mode, and (E) and (F) in the second slowest mode, respectively. The line plot connecting the points that indicate the correlation values is used to guide the eye.

The residue fluctuations in the slowest two modes of p1-p6_D30N_, p1-p6_D30N/N88D_, and ^LP1′F^p1-p6_D30N/N88D_ variants are compared with those of p1-p6_WT_ (Fig.3). The correlation coefficient values between the fluctuation vectors of each residue in the two respective structures indicate the orientational (directional) variation in their fluctuations in the given mode. With the protease mutations, the largest orientational difference in the fluctuations is observed mostly at the proximity of residue 69 in the unprimed monomer and at residues 56, 78, 93 in both monomers in the slowest mode (Fig.3A). In the second slowest mode, the fluctuations of residues 25, 26, 27, 39 in the unprimed monomer and residues 16, 49, 50, 51, 97 in both monomers display largest orientational difference (Fig.3D). These residues lie at the hinge axes of rotational motion identified by the residue mobility profiles in Fig.2.

The correlation coefficients of the residue fluctuation vectors between p1-p6_WT_ and ^LP1′F^p1-p6_D30N/N88D_ in both modes are in general higher than they are between p1-p6_WT_ and p1-p6_D30N/N88D_. To quantify, when the residues with maximum variations in their orientational correlations that are below the lower standard deviation bound (average minus one standard deviation) are considered, the average correlation coefficient value which is 0.61 between p1-p6_WT_ and p1-p6_D30N/N88D_ increases to 0.90 between p1-p6_WT_ and ^LP1′F^p1-p6_D30N/N88D_ in the slowest mode. Similarly, in the second slowest mode, the average correlation coefficient value between p1-p6_WT_ and p1-p6_D30N/N88D_ is 0.63, whereas it is 0.69 between p1-p6_WT_ and ^LP1′F^p1-p6_D30N/N88D_. In both modes, the correlation of p1-p6_D30N_ with p1-p6_WT_ is even less than that of p1-p6_D30N/N88D_ which has the signature mutations of nelfinavir resistance that occur in association with p1-p6 cleavage site mutations (Kolli et al. 2009).

The residues that display the maximum variations between the directions of fluctuations can also be visualized on the structures in Fig.3, as color coded according to the change in the correlation values of p1-p6_D30N/N88D_ (Fig.3B,E) and ^LP1′F^p1-p6_D30N/N88D_ (Fig.3C,F) compared to p1-p6_WT_. In the slowest mode, the higher correlation values of residues 67, 68, 69, 56′, and 78′ between ^LP1′F^p1-p6_D30N/N88D_ and p1-p6_WT_ structures compared to those between p1-p6_D30N/N88D_ and p1-p6_WT_ structures are evident (Fig.3B,C). On the other hand, the highest increase in the correlation values with p1-p6_WT_ structure as a result of coevolution is observed for residues 25, 26, 27, and 39 in the second slowest mode (Fig.3E,F).

The correlated mutations at residues 30 and 88 lead to a significant change in the orientation of the fluctuations of residue 69 in the slowest mode, which leads to the dramatic difference in the dynamic behavior with an additional asymmetry between the two monomers of the protease. The other significant changes in the fluctuation orientation in this mode are at residues 56′ and 78′. Nevertheless, with the coevolving mutation at P1′ site in the substrate, the re-orientation of the fluctuations of these residues close to their wild-type position is observed remarkably (Fig.3A–C). Residues 56 and 78, being flap and substrate cleft residues with low mobility, are found at the hinge region that connects the 40's and 70's loops to the flaps, respectively. Residue 69 is found on the 70's loop which moves in a manner of a cantilever with the flaps, where flaps close as the cantilever moves up (Lebon and Ledecq 2000). Therefore, the motion of residues 56, 69, and 78 is coupled to the motion of the flaps, which is known to be the significant functional motion of the protease (Nicholson et al. 1995; Kurt et al. 2003; Hornak and Simmerling 2007). The importance of such protease regions which interact with the flaps is implied in studies of developing allosteric inhibitors for HIV-1 protease that do not compete for the active site, where they are targeted as allosteric sites (Lebon and Ledecq 2000; Perryman et al. 2004; Hornak and Simmerling 2007; Yang et al. 2012). On the other hand, in the second slowest mode, protease mutations lead a fluctuation orientation difference particularly at residue 39 in the unprimed monomer together with the active site and dimerization interface residues, which is recovered by the coevolving mutation on the substrate (Fig.3D–F). Residue 39 has high mobility in the slowest mode yet acts as a hinge in the second slowest mode, where it interacts and fluctuates in the opposite direction with the flaps of the same monomer (Ozer et al. 2010). Due to this anticorrelated behavior with the flap motion, 39 is also an important residue which has been considered as a potential allosteric inhibition site (Tozzini and McCammon 2005). By introducing mutations and specific cross-links at residues around 39 to restrict the hydrophobic core rearrangements, the essential role of core flexibility in modulating the activity of HIV-1 protease has been demonstrated (Mittal et al. 2012).

The most variation being in the orientation of the fluctuations around the hinge axes implies that the protease mutations perturb the mechanistically crucial sites that mainly coordinate the intrinsic dynamics of the protease in interaction with its substrate. The protease mutations with the example of p1-p6 substrate complex here show the deformation of the hinge axes, representing a global deformation with local perturbations. Nevertheless, the fluctuations in the coevolved structure have the highest correlation with that of the wild-type structure among other mutant structures. The deformation in the direction of fluctuations and the additional asymmetry of the protease monomers is recovered considerably by this coevolution. This provides a more comprehensive view with dynamics incorporated as an additional dimension, rather than just the structural information about the positions of the mutations.

The structures of p1-p6 bound protease studied here are the representative conformations of the largest of five, three, and four clusters in p1-p6_WT_, p1-p6_D30N/N88D_, and ^LP1′F^p1-p6_D30N/N88D_ MD simulation trajectories, respectively (Table1). Then, the ANM analyses of the representative members of the second largest clusters are also investigated. In the most cooperative modes of motion of these structures, the maximum orientational variations are observed in the same residues that lie along the hinge axes in p1-p6_WT_ where the directions of their fluctuations are significantly distorted in p1-p6_D30N/N88D_. On the other hand, these variations in the fluctuation directions of the hinge residues of ^LP1′F^p1-p6_D30N/N88D_ largely remain within the variations observed in p1-p6_WT_ compared to those observed in p1-p6_D30N/N88D_.

#### Coevolution in NC-p1

**Figure 4:**
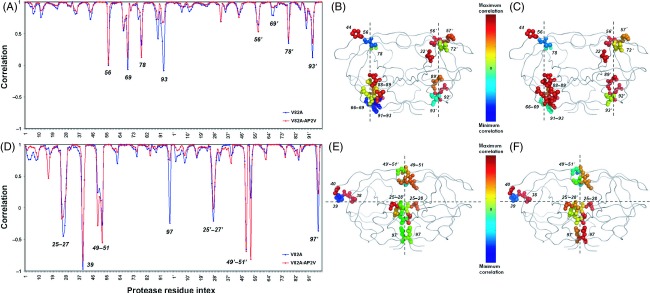
Orientational correlation of protease residues' fluctuations of NC-p1_V82A_ and ^AP^^2^^V^NC-p1_V82A_ to those of NC-p1_WT_ in the first (A) and second (D) slowest modes. The residues that display the maximum variations between the directions of fluctuations are color coded according to the change in the correlations of NC-p1_V82A_ and ^AP^^2^^V^NC-p1_V82A_ compared to NC-p1_WT_; (B) and (C) in the first slowest mode, and (E) and (F) in the second slowest mode, respectively. The line plot connecting the points that indicate the correlation values is used to guide the eye.

Figure4 displays the orientational correlation values of the fluctuation vectors of protease residues of NC-p1_V82A_ and ^AP2V^NC-p1_V82A_ with respect to NC-p1_WT_ for the slowest two modes. The residues of the protease mutant that display the largest orientational difference in their fluctuations with respect to the wild type are 56, 69, 78, 93 in both monomers in the slowest mode (Fig.4A). In the second slowest mode, the fluctuations of residue 39 in the unprimed monomer and residues 25, 26, 27, 49, 50, 51, 97 in both monomers display the largest orientational difference as a result of the protease mutation (Fig.4D). These residues with largest deviations in their orientations lie along the hinge axes of rotational motion in both modes (Fig.2), and they are found at almost identical sites in the p1-p6 and NC-p1 bound structures, where they interact with functional regions such as flaps and cleft covering the active site.

The correlation coefficients of the residue fluctuation vectors between NC-p1_WT_ and ^AP2V^NC-p1_V82A_ in both modes are higher than they are between NC-p1_WT_ and NC-p1_V82A_, yet the increases in the correlations are not as significant as in p1-p6. By considering the residues with maximum variations in their orientational correlations that are below the lower standard deviation bound (average minus one standard deviation), it is assessed that the average correlation coefficient value which is 0.45 between NC-p1_WT_ and NC-p1_V82A_ increases to 0.58 between NC-p1_WT_ and ^AP2V^NC-p1_V82A_ in the slowest mode. In the second slowest mode, the average coefficient value does not improve much (0.026–0.052, respectively) for the correlations of NC-p1_WT_ with NC-p1_V82A_ and ^AP2V^NC-p1_V82A_. The relatively low increase in the average correlation values is due to the opposing behavior observed in the residues as a result of coevolution; some correct their fluctuations while some fluctuate more diversely with respect to the wild type.

The structures in Fig.4 display the residues that exhibit the maximum variations between the directions of fluctuations, as color coded according to the change in the correlation values of NC-p1_V82A_ (Fig.4B,E) and ^AP2V^NC-p1_V82A_ (Fig.4C,F) compared to NC-p1_WT_. In the slowest mode, the correlations of residues 66, 69, 89, 92, 93, 77′, 89′, and 93′ between ^AP2V^NC-p1_V82A_ and NC-p1_WT_ are higher than their correlations between NC-p1_V82A_ and NC-p1_WT_ (Fig.4B,C). In the second slowest mode, an increase in the correlation values with NC-p1_WT_ as a result of coevolution is observed for residues 25, 26, 27, 28, and 97 of both monomers, whereas the correlation values of residues 49, 50, and 51 on the flap regions decrease (Fig.4E,F).

Additionally, the protease mutation at residue 82 causes the difference in the dynamic behavior with an additional asymmetry between the protease monomers, particularly by the change in the orientation of the fluctuation of residue 93 in the slowest mode and residue 97 in the second slowest mode. The deformed orientations of the residue fluctuations return closer to their behavior in the wild-type dynamics by the consequent coevolving mutation at P2 site in the NC-p1 substrate (Fig.4). The importance of the dimerization interface regions was also emphasized in allosteric inhibition studies of HIV-1 protease: Besides the allosteric inhibition studies targeting the flap motion of HIV-1 protease, another class of allosteric inhibitors investigated are dimerization inhibitors that would prevent the formation of the active protease homodimer by binding to the dimerization interface (Hornak and Simmerling 2007; Yang et al. 2012).

The structures of NC-p1 bound protease studied here are the representative conformations of the largest of four, three, and five clusters in NC-p1_WT_, NC-p1_V82A_, and ^AP2V^NC-p1_V82A_ MD simulation trajectories, respectively (Table1). As in p1-p6 bound structures, the representative members of the second largest clusters are also investigated by ANM. The residues that lie along the hinge axes affirm maximum orientational variations with significant distortions in the directions of their fluctuations in NC-p1_V82A_ in the most cooperative modes of motion. Furthermore, the variations in the fluctuation directions of the hinge residues of ^AP2V^NC-p1_V82A_ remain within the variations observed in NC-p1_WT_ compared to those observed in NC-p1_V82A_.

Here, within the NC-p1 structures in the slowest two modes, higher correlation is observed in most of the hinge residues between wild-type and coevolved complex structures compared to that between wild-type and V82A mutant, resembling the orientational correlations within the p1-p6 structures. Yet, the lower correlations between the wild-type and the coevolved structures of NC-p1 compared to those in the p1-p6 structures, as well as the decreasing correlations for some of the hinge residues, suggest that the repossession of the structural dynamics as a result of the re-orientation by the coevolutionary mutation in the NC-p1 site is not as strong as that in the p1-p6 site.

### Coevolved NC-p1 and other substrate complex structures

**Figure 5:**
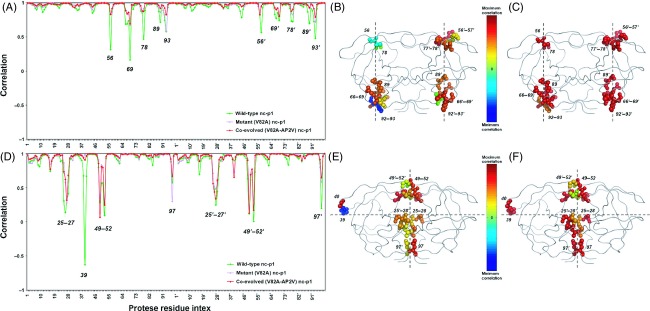
Orientational correlation of protease residues' fluctuations of NC-p1_WT_, NC-p1_V82A_, and ^AP^^2^^V^NC-p1_V82A_ to those of the averaged wild-type natural substrate complexes other than NC-p1 in the first (A) and second (D) slowest modes. The residues that display the maximum variations between the directions of fluctuations are color coded according to the change in the correlation of NC-p1_WT_ and ^AP^^2^^V^NC-p1_V82A_ compared to the averaged wild-type natural substrate complexes other than NC-p1; (B) and (C) in the first slowest mode, and (E) and (F) in the second slowest mode, respectively. The line plot connecting the points that indicate the correlation values is used to guide the eye.

The dynamics of mutant NC-p1 complex structures are also studied with respect to that of the wild-type structures of HIV-1 protease bound to the natural substrates other than the NC-p1 itself, namely MA-CA, CA-p2, p2-NC, p1-p6, RT-RH, and RH-IN. The orientational correlations of the fluctuation vectors of protease residues of the NC-p1 complex structures with those of the wild-type complex structures of each of the other six natural substrates are calculated separately, and the average correlation per residue position over six correlation values is computed. The orientational correlation of protease residues of NC-p1_WT_, NC-p1_V82A_, and ^AP2V^NC-p1_V82A_ with respect to the average of the wild-type structures of the other six natural substrates in the two most cooperative modes of motion is displayed in Fig.5. The residues that display the maximum orientational difference in their fluctuations are observed at the similar hinge regions as in the previous cases, specifically at proximity of residues 56, 69, 78, 89, and 93 of both monomers in the slowest mode (Fig.5A), and at residues 39 of the unprimed monomer, 97 of the primed monomer, and 25, 26, 27, 28, 52 of both monomers in the second slowest mode (Fig.5D). The importance of these hinge regions in both function and allosteric communication, being either residues of the active site (25–28), the flaps (52, 56), the substrate cleft (78, 89), the dimerization interface (93, 97), or those exhibiting linked motion with the functionally important flaps (39, 69), should be noted (Lebon and Ledecq 2000; Perryman et al. 2004; Tozzini and McCammon 2005; del Sol et al. 2006; Hornak and Simmerling 2007).

In both modes, the correlations of the residue fluctuation vectors of the hinge regions in each of the natural substrate complexes other than NC-p1 with both of the mutant NC-p1 complex structures are higher compared to those with the wild-type NC-p1 complex structure. The correlation coefficients calculated over the least correlating residues (having correlation values below the lower standard deviation bound) are 0.64, 0.84, and 0.87 in the slowest mode and 0.32, 0.59, and 0.57 in the second slowest mode in turn for NC-p1_WT_, NC-p1_V82A_, and ^AP2V^NC-p1_V82A_ with respect to the average of the wild-type structures of the other six natural substrates. It appears that the correlation between the NC-p1 complex structure with the other natural substrate bound structures increases as a result of the mutation V82A in the protease. However, with the consecutive coevolving mutation AP2V in the substrate, this correlation does not increase significantly yet decreases slightly in the second slowest mode.

The residues with maximum orientational differences between their directions of fluctuations can also be observed on the structures in Fig.5, as color coded according to the change in the correlation of the NC-p1_WT_ (Fig.5B,E) and ^AP2V^NC-p1_V82A_ (Fig.5C,F) compared to the average of the wild-type protease structures bound to the other six natural substrates. As a result of the mutations in the protease and the NC-p1 substrate, the residues of the hinge regions explicitly mentioned above exhibit increased correlations with the rest of the natural substrate bound protease structures.

Here, it is interesting to note the higher number of protease residues in NC-p1_V82A_ and ^AP2V^NC-p1_V82A_ possessing higher correlations with those in the wild-type complex structures bound to the rest of the natural substrates. This is consistent with the structural rationale for HIV-1 protease binding to the NC-p1 cleavage site given in Prabu-Jeyabalan's work (Prabu-Jeyabalan et al. 2004), where they solved the crystal structures of wild-type and V82A mutant proteases in complex with their respective wild-type and AP2V mutant NC-p1 substrates. They observed that the AP2V mutant peptide bound the mutant protease more optimally than the wild-type NC-p1 peptide bound the wild-type protease. That is, the AP2V mutation on the peptide coevolving with the V82A mutation on the protease re-orients the peptide to a conformation which is more similar to those of the other natural substrate-protease complexes than the NC-p1 (Prabu-Jeyabalan et al. 2004).

In the analyses outlining the coupling between catalysis and conformational mechanics, there is growing evidence that enzymatic activity results from a delicate interplay between chemical kinetics and molecular motions (Yang and Bahar 2005). The catalytic sites are found at proximity of binding sites which enjoy flexibility to accommodate the ligand binding, and the accompanying large-scale conformational changes are connected to the hinge motion (Ferreiro et al. 2011). Overall, the variation in the residues at the hinge regions of HIV-1 protease in the functional modes is required for the protease to process different substrates, which results in specific cleavage rates for the proper functioning of the virus life cycle. Therefore, the interdependent nature of the substrate recognition allowing the protease to recognize various nonhomologous sequences as natural substrates may be partly due to this adaptability in the hinge regions which are the mechanistically crucial sites that mainly coordinate the intrinsic dynamics.

## Conclusion

Structural dynamics analyses contribute largely to the understanding of functional and evolutionary properties of proteins, which suggest that the preservation of dynamic properties is critical for maintaining the biological function (Gerek et al. 2013). The global motions of the proteins are described by the most cooperative normal modes of the ANM. These modes have the highest contribution to the flexibility profiles, and they are predominantly defined by the proteins' architecture. Therefore, they are generally functionally relevant, evolutionarily conserved and are more robust with respect to mutational perturbations (Liu and Bahar 2012). Also, the conserved hinge regions identified in these conserved modes are shown to play decisive roles in conformational transitions induced by binding. Here, the examination of the structural and dynamic properties of the mutant and coevolved structures of p1-p6 and NC-p1 substrate complexes contributes to the understanding of the binding as well as the drug-resistant mechanism of HIV-1 protease. Overall, there seems to be interplay between the variation in the fluctuations of the important hinge regions and the variation in the substrate sites of the protease in regard to functionality. That is, a plausible complex dependence between the hinge behavior and the specific functionality of the substrate with respect to the rate of cleavage can be inferred. The mutation in the substrate allows the protease residues to re-orient and thus fluctuate as in the functional conformation, and justify the existence of this coevolutionary mutation for the conservation of, at least, the fluctuations and flexibility.

Understanding the determinants of ligand recognition and binding in sequence, structure, function, and dynamics paradigm is now a major challenge in drug discovery. The structural data for target proteins with different ligands display the contribution coming from both partners in selecting the bound forms. The intrinsic dynamics of proteins appears to be optimized by evolution for functional interactions, yet the ligand selects the one that best fits its structural and dynamic properties among the conformations accessible to the unbound protein. In binding, the variety of ligands with different compositions and shapes as well as affinity and selectivity can be explained by the conformational flexibility of receptors. Being related to dynamics and evolution, flexibility should also have impacts on structural divergence connected to the orientational difference in the fluctuation of the molecules. This implies that drug-resistant studies should go beyond the concept of inhibition of structure to the concept of inhibition of functional dynamics by focusing on flexibility as well.
